# Searching for a cure on Facebook: Patterns of social media use amongst caregivers of children with brain tumors

**DOI:** 10.1002/cam4.4693

**Published:** 2022-03-28

**Authors:** Tyler T. Miller, Scott H. Maurer, James T. Felker

**Affiliations:** ^1^ University of Pittsburgh School of Medicine Pittsburgh Pennsylvania USA; ^2^ Department of Pediatrics, Division of Hematology/Oncology University of Pittsburgh Pittsburgh Pennsylvania USA; ^3^ Division of Palliative Medicine and Supportive Care UPMC Children's Hospital of Pittsburgh Pittsburgh Pennsylvania USA

**Keywords:** brain tumor, caregivers, communication, neuro‐oncology, parents, social media

## Abstract

**Objective:**

Social media (SM) is ubiquitous in modern society. How SM provides information, advice, and community to families coping with childhood brain tumors is poorly understood. We sought to understand how caregivers of children with brain tumors use and are affected by SM.

**Methods:**

A survey was administered to caregivers of children who were receiving or within the last 5 years received chemotherapy for pediatric brain tumors. Differences in variables across groups were evaluated using nonparametric tests and chi‐square tests.

**Results:**

Thirty‐five of 36 caregivers acknowledged use of SM. Facebook was the most used platform (86%). Fifty‐eight percent and 47% used SM to read and share information about their child's cancer, respectively. Thirty‐four percent were comforted while 40% were bothered by cancer‐related information on SM. Eleven participants (31%) sought a second opinion based on information from SM. Caregivers of children with a poor prognosis were more likely to use a treatment from SM that was not initially recommended by their oncologist (*p* = 0.043).

**Conclusion:**

SM is commonly used by caregivers to obtain and share care‐related information. Many noted positive and negative effects of SM on emotional wellness. SM influenced treatment decisions, and this effect was stronger with poorer prognosis. Our results demonstrate the dichotomous impact of SM in medicine—it is a source of both solace and anxiety, a place to confirm treatment decisions and to create doubt in the treatment decisions of the oncologist. This illustrates the importance of discussing SM with caregivers of children with brain tumors.

## Graphical abstract

We sought to understand how caregivers of children with brain tumors use and are affected by SM by administering a survey to 36 caregivers of children who were receiving or within the last 5 years received chemotherapy. Many noted positive and negative effects of SM on emotional wellness. SM influenced treatment decisions, and this effect was stronger with poorer prognosis.

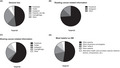

## 1 INTRODUCTION

Social media (SM) connects billions of people worldwide. For many, it has become integrated with daily life.^1^, ^2^ Patients and their caregivers (parents, grandparents, guardians, etc.) look to SM for information, advice, and support. Personal posts, comments, and support groups are all ways that SM users communicate.^3^, ^4^, ^5^ In the field of pediatric oncology, emotions, decisions, and well‐being all have the potential to be influenced by SM.

SM platforms such as Facebook™ and Twitter™, as well as patient‐focused websites such as PatientsLikeMe™ and CaringBridge™, are platforms caregivers use to exchange information and support.^6^, ^7^ Parents of children with cancer consume online material related to their child's diagnosis because they find it easy‐to‐access and informative.^8^ Adult oncology patients similarly rate the internet as an important tool for finding information related to their cancer.^9^ Despite these benefits, a significant amount of information reported on social media platforms is not scientifically accurate,^10^, ^11^ and it can be difficult for patients and their family to discern the quality of the information they find. There is also evidence that online health information can lead patients to alter treatment, and that social media has varying beneficial and harmful effects on patient well‐being, sense of social support, and the patient–doctor relationship.^4^, ^5^, ^12^, ^13^ In pediatric oncology, most caregivers report use of SM to access information related to their child's cancer.^3^ What is not known is how information on SM affects the well‐being and treatment decisions of families coping with childhood cancer.

Families coping with pediatric brain tumors represent a population faced with uniquely difficult decisions, psychosocial stress, and long‐lasting consequences for the patient and their family.^14^, ^15^ Understanding SM's influence may allow physicians to help families use social media as a positive tool and preemptively counsel them regarding potential issues they may encounter with SM. We conducted a cross‐sectional, quantitative survey of caregivers of children with brain tumors. Our primary goal was to evaluate the scope of social media use among this population and gain understanding of its influence on caregivers in pediatric oncology.

## 2 METHODS

Subjects were recruited during outpatient pediatric oncology visits occurring at UPMC Children's Hospital of Pittsburgh. Subjects were caregivers of children with central nervous system (CNS) malignancy on therapy or within the first 5 years of post‐treatment monitoring. Subjects were ineligible if they were under the age of 18 or a caregiver for patient over 21 years of age. Permission to approach potential subjects was received by the child's treating oncologist. The enrollment process, survey, and methodology for this study were approved by the Institutional Review Board of the University of Pittsburgh. Informed consent was obtained from all individual participants included in the study.

After obtaining written, informed consent, an investigator not involved in the patient's medical care administered a‐47 item questionnaire via a tablet computer to the caregiver. The investigator remained present to answer any questions about the survey. The survey focused on the caregiver's SM usage, demographics, frequency and type of SM use, and any changes to treatment based on information received on SM. Five‐point Likert scales (with choices including strongly disagree, disagree, neutral, agree, and strongly agree) were used to measure attitudes toward SM. Some questions were automatically skipped if, based on previous answers, they did not apply to the caregiver. All surveys were completed between August 2019 and February 2021.

After collecting all surveys, patients' charts were reviewed for information related to diagnosis and treatment. Descriptive statistics were reported using mean with standard deviation for the Likert scale items/questions and frequencies with percentiles for the multiple‐choice items/questions. Mann–Whitney *U* test was used to compare Likert scale items/questions between groups. Chi‐Square test was used to compare categorical items/questions between groups. Statistical significance was defined as *p* < 0.05. All statistical analyses were performed using SPSS V24.0 (IBM Corp.). A secondary analysis was done to compare results between patients with “fair prognosis” versus “poor prognosis.” Once patients' diagnosis and treatment regimen were confirmed using their charts, patients' primary oncologist judged their 5‐year overall survival and discussed this with investigators. Those with cancer whose 5‐year overall survival was estimated at <5% were classified as “poor prognosis.” All others were categorized as “fair prognosis.”

## 3 RESULTS

Thirty‐six caregivers enrolled in our study and completed the survey. Four caregivers were approached but declined participation. During the pandemic, five caregivers began the survey online at home, and three of these five completed the survey to be included in the results. Participants were mostly white (86%) mothers (94%) with an average age of 38 years (*SD* = 10.3) (Table 1). The average patient age was 9 years (*SD* = 5.3), and the most common diagnosis was a low‐grade glioma (42%). Twenty‐nine caregivers (81%) self‐reported at least a moderate level of anxiety (Table 1).

**TABLE 1:** Patient Characteristics

A. Demographics		
Variable	*n*	%
Relationship to patient		
Mother	33	92%
Father	2	6%
Grandmother	1	3%
Age		
20–29	5	14%
30–39	17	47%
40–49	11	31%
50–59	2	6%
60–69	1	3%
Race		
Asian	1	3%
Black	3	8%
White (Hispanic)	1	3%
White (Non‐Hispanic)	31	86%
Education		
No/some high school	1	3%
High school diploma/ GED	18	50%
Associate's degree	2	6%
Bachelor's degree	11	31%
Post‐bachelor's degree	4	11%
Annual household income		
Below $25,000	7	19%
25,000–50,000	6	17%
50,000–75,000	8	22%
75,000–100,000	3	8%
Above $100,000	12	33%
Patient's age		
0–5	13	36%
6–10	8	22%
11–15	9	25%
16–20	6	17%
Patient's sex		
Male	21	58%
Female	15	42%
Patient's diagnosis		
Atypical Teratoid Rhabdoid Tumor (ATRT)	1	3%
Diffuse Intrinsic Pontine Glioma (DIPG)	2	6%
Ependymoma	2	6%
Germinoma	1	3%
High‐grade glioma (HGG)	3	8%
High‐grade neuroepithelial tumor	1	3%
Low‐grade glioma (LGG)	15	42%
Medulloblastoma	3	8%
Pineoblastoma	2	6%
Primitive neuro‐ectodermal tumor (PNET)	1	3%
Relapsed ATRT	1	3%
Relapsed Ependymoma	4	11%

B. Self‐reported social media usage and anxiety		
Variable	*n*	%
Frequency of SM use		
Never	1	3%
Rarely	3	8%
Weekly	4	11%
Daily	14	39%
Several times per day	14	39%
Type of SM use		
General use	32	89%
Reading cancer‐related information	21	58%
Sharing cancer‐related information	17	47%
Frequency of reading info on SM		
Rarely	4	19%
Weekly	10	48%
Daily	4	19%
Several times per day	3	14%
Frequency of sharing info on SM		
Rarely	6	35%
Weekly	10	59%
Daily	1	6%
Several times per day	0	0%
Caregiver anxiety level		
Not at all	0	0%
A little	7	19%
Moderate	17	47%
Very	7	19%
Extreme	5	14%

Of the 36 participants, 35 (97%) acknowledged use of SM. Most (78%) noted at least daily use. In addition to general use of SM, many caregivers reported using SM to read about their child's cancer (58%) and to share information (47%) about their child's care. Only 33% and 6% read and shared such information, respectively, on a daily basis (Table 1).

**FIGURE 1:**
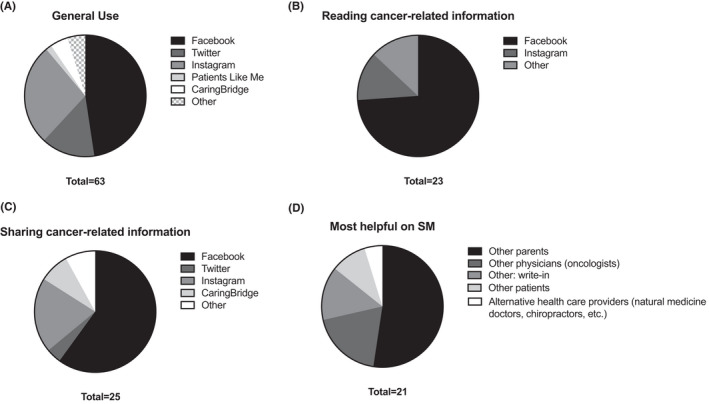
SM platforms and helpful users. Total distribution of participants' used platforms is shown for “General Use” (A), “Reading Cancer‐related Information” (B), and “Sharing Cancer‐related Information” (C). Total distribution of who was most helpful on SM (D) is also shown. Participants chose more than one response if applicable

Of those who used SM, Facebook (86%) was the most used SM platform, followed by Instagram (49%) (Figure 1). These sites were used not only for general SM use, but also to read and share information about their child's cancer. Answers written in the “Other” section of Figure 1 included “Momcology” and “Inspire” as sites used to read and share information. When asked who was most helpful on SM, 52% responded “other parents” (Figure 1).

**FIGURE 2:**
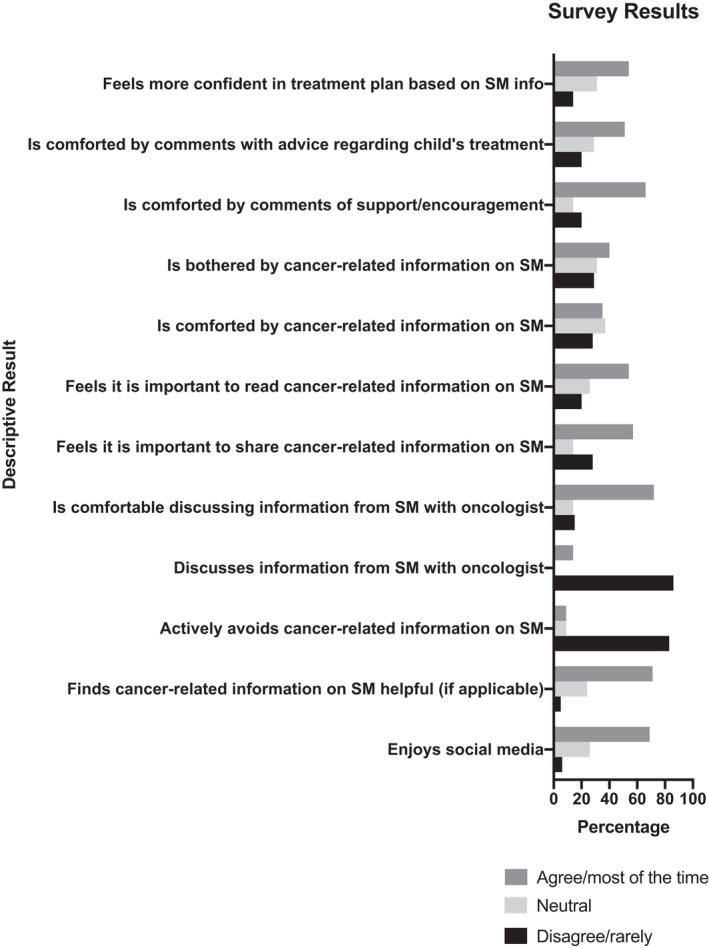
Descriptive survey results. Results were stratified based on Likert scale responses (1–2 = disagree/rarely, 3 = neutral, and 4–5 = agree/most of the time)

**FIGURE 3:**
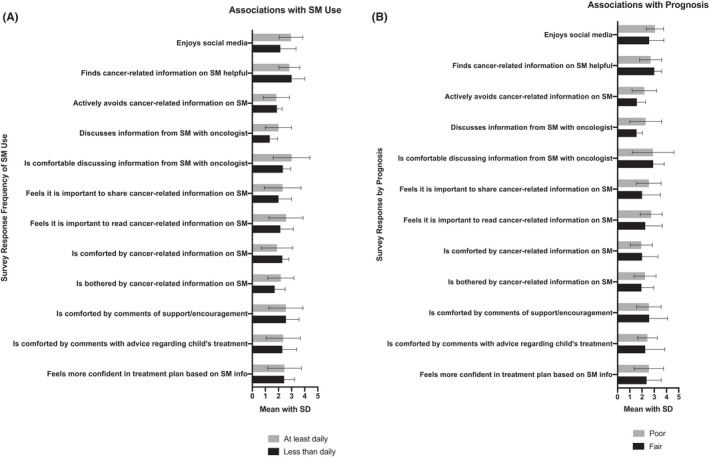
Descriptive survey results grouped by (A) frequency of SM use and (B) anticipated prognosis of child. Results were listed as mean with standard deviation, with 5 representing the most positive response (agree/always) and 1 being disagree/never

Thirty‐four percent of SM users felt comforted by cancer‐related information they read on SM, 40% indicated such information on SM was bothersome, and 60% noted actively avoiding cancer‐related information, at least occasionally (Figure 2). As many as 38% never discussed the information they read online with their oncologists and 14% noted that they were uncomfortable discussing it (Figure 2). Eleven participants (31%) sought a second opinion based on information from SM, and four (11%) children received a treatment found on SM which was not initially recommended by their oncologist. Treatments received by children that were found on SM included proton radiation, sodium thiosulfate, clinical trials, and surgical intervention. The primary oncologists had been made aware of the treatments in all four cases. They were approving of the treatments in three out of the four cases, per caregiver responses. The frequency of SM use was not significantly associated with demographics or other Likert scale responses (Figure 3). Self‐reported anxiety levels were also not significantly associated with demographics or other Likert scale responses.

As part of a secondary analysis, there were 23 patients considered to have a “fair” prognosis and 13 considered to have a “poor” prognosis. Poor prognosis was significantly associated with the use of a treatment found on SM that was not initially recommended by an oncologist (*p* = 0.043). There were no significant differences between the groups regarding anxiety levels, enjoyment of social media, and seeking a second opinion (Figure 3).

## 4 DISCUSSION

Our data describe patterns of SM use, feelings related to SM, and SM‐influenced decisions among caregivers of children with brain tumors. SM use was ubiquitous among our study population, and caregivers noted mixed feelings regarding whether it was emotionally beneficial or harmful. We demonstrate that information found on SM can influence patient care, especially in patients with poor prognosis. These data may indicate an opportunity for pediatric oncology providers to positively influence SM responses in caregivers either by addressing SM use directly with caregivers or via improving the quality of SM content through their own SM use.

The generalizability of these findings may be limited by our study's focus on a specific and vulnerable population of caregivers of children with brain tumors; however, in the face of the rapid expansion in content and popularity of SM, these findings seem congruent with emerging data on the broader population of caregivers of children with cancer. SM use among adults has risen from 5% in 2005 to 79% in 2019.^2^ This global expansion in content and popularity is likely to rise in the COVID‐19 and post‐COVID‐19 era,^16^ and parents of children with cancer have indicated that SM provides quick access to information^8^ along with support from others.^17^, ^18^ Congruently, our sample demonstrated near universal (97%) and frequent (78% at least daily) use of SM despite a broad distribution of education level and household income. A majority of caregivers in our sample participated in SM not only for general use, but also for reading information about their child's cancer. Foot et al. recently demonstrated similar, though less frequent (41% at least daily use), patterns of SM use among a group of parents of children with cancer who ranged from being close to diagnosis to well into survivorship.^18^ Based on the rapid expansion of information and social support on SM, along with the consistent use of SM by caregivers across studies and demographics, our data seems to indicate that caregivers of children with brain tumors may behave similarly to other families and providers in pediatric oncology.

SM users in our sample largely enjoyed SM, especially those who used it frequently. Their stances on cancer‐related SM posts and comments were more varied. A third of SM users found cancer‐related information on SM comforting and 40% found it bothersome. Opposing responses to SM, while possibly due to differences in personality, may also be due to the perceived positivity or negativity of one's encounters on SM.^19^ Whatever the case, when faced with information that affects their child's life, many caregivers were able to identify an emotional response attached to it.

SM has been found to affect patient adherence to treatment and parent's attitudes toward medical interventions,^20^, ^21^ and our findings support the idea that SM may influence childhood cancer treatment. Eleven caregivers sought a second opinion based on SM while four children received treatment found on SM which was not initially recommended by their oncologist. Our study did not investigate the route or reasoning by which caregivers sought a second opinion, but it may be related to findings on SM which diverge from their oncologist's plan. Additionally, caregivers of children with poor prognoses were significantly more likely to act on material from SM, using treatments from SM more frequently. Caregivers of patients with brain tumors like diffuse intrinsic pontine glioma (DIPG), with a near 0% long‐term survival, understandably may have increased distress or feelings of desperation leading to these caregivers' willingness to act on information from SM. If true, SM has the potential to sow doubt in the patient–physician relationship at a time when that bond is most important. While our study was not powered to find a significant link between prognosis and caregiver anxiety level, a large, multicenter, prospective investigation could find associations of increased distress related to SM use. Qualitative data would be useful in future studies as it may also give finer detail regarding these feelings.

Recognition of the potential influence of SM creates an opportunity for pediatric oncology providers to counsel caregivers regarding the positive aspects of SM communities as well as the hazards of potentially false information online and the importance of evidence‐based care for their child. With a variety of information online that is easy‐to‐access at any time, further investigation by caregivers is to be expected, yet these examples reinforce the importance of the patient–doctor relationship. Ensuring that patients' families feel comfortable discussing information they find online with their physician substantiates the value that a parent brings to the care of their child and facilitates trust. Preemptive conversations regarding SM use may be initiated to advise caregivers about what they may or may not find on SM, how they may sometimes find comfort in talking to others about their child's cancer, that they may be distressed by things they read, and that they may see things on SM of varying quality which may lead them to second guess shared treatment. By discussing SM with parents in an open way, pediatric oncology providers create a space where families can make complex decisions with important factors in mind—relationships, experiences, and beliefs.^22^ Ideally, open and nonjudgmental dialogue about information, a parent has found on SM (or elsewhere) would serve to validate the parent's effort to advocate for their child and deepen the therapeutic relationship.^23^

Implementing a form of education for caregivers on the benefits and risks of SM would help them effectively judge information. Anticipatory guidance early in the oncologist‐caregiver relationship, as well as clear willingness to discuss things parents find online, could be immensely helpful to caregivers. Infographics or pamphlets may be one way to help caregivers navigate SM, or simply asking at every visit, “would you like to discuss anything you have been reading on social media?” may be another. Physician activity on SM, including the practice of spreading evidence‐based medicine and refuting misinformation, is also a way to disseminate medical knowledge and help the public receive reliable information.^24^, ^25^, ^26^ Providing personalized medical advice can come with ethical and legal concerns, so spreading good and easy‐to‐interpret evidence while respecting the protections that exist for patients is crucial.^27^, ^28^ SM platforms may even be adapted for communication among patients, providers, and organizations.^28^, ^29^, ^30^ One important option is the initiation of online support groups that allow caregivers to ask questions, share stories, and comfort one another. Some studies have shown the effectiveness of online support groups at improving the well‐being of caregivers of sick children.^31^, ^32^ As a whole, increasing access to support groups and accurate information would likely improve SM users' online experiences and promote quality care.^29^, ^33^

## 5 STUDY LIMITATIONS

Limitations of this study include its small sample size, which was restricted due to the specific inclusion criteria. Inclusion criteria specified a limited range of diagnoses in order to focus our description of SM use on a highly vulnerable and important population of caregivers in pediatric neuro‐oncology. COVID‐19 became prevalent after the first 20 surveys were administered, and a few of the subsequent surveys were administered virtually. We did an analysis which showed that the use of SM before and after COVID‐19 did not change. Most of our respondents were mothers, and our data does not adequately assess the potentially different paternal perspective of SM use. The lack of racial and ethnic variability in this population limits the external validity of these results among other groups, where there may be different patterns of SM use. Future studies could benefit from seeking respondents from all genders and racial/ethnic backgrounds. Additionally, although surveys were administered by researchers unknown to the patients, recruitment of participants was done by their treating physicians, which increases the possibility of selection bias and may have made some participants less likely to disclose personal information or treatment alterations. All data were self‐reported.

## 6 CLINICAL IMPLICATIONS

These results demonstrate the dichotomy of SM in medicine—it can be a source of both solace and anxiety. It can both be a tool that empowers families, and it can act as a wedge that disrupts the doctor‐family relationship. Prospective studies of SM use by parents of children with brain tumors may help us better understand the impact of SM on their care. In the meantime, pediatric oncology providers may have an opportunity to positively influence caregiver reactions to social media by providing anticipatory guidance about SM use to their patients and families.

## 7 CONCLUSIONS

SM is commonly used by caregivers of children with brain tumors to obtain and share care‐related information, and its use is likely to rise as younger generations become parents. Many caregivers noted positive and negative effects of SM on emotional wellness. SM affected treatment decisions, and this effect was stronger with poorer prognosis.

## CONFLICT OF INTEREST

The authors have no relevant financial or nonfinancial interest to disclose.

## AUTHOR CONTRIBUTION

All authors contributed to the study conception and design. Material preparation, data collection, and analysis were performed by Tyler Miller, Scott Maurer, and James Felker. The first draft of the manuscript was written by Tyler Miller and all authors commented on previous versions of the manuscript. All authors read and approved the final manuscript.

## ETHICS

The survey and methodology for this study was approved by the Human Research Ethics committee of the University of Pittsburgh (Ethics approval number: 19050087).

## Data Availability

The data that support the findings of this study are available from the corresponding author upon reasonable request.
